# Effectiveness of the Dader Method for pharmaceutical care in patients with bipolar I disorder: EMDADER-TAB: study protocol for a randomized controlled trial

**DOI:** 10.1186/1745-6215-15-174

**Published:** 2014-05-20

**Authors:** Andrea Salazar-Ospina, Pedro Amariles, Dora M Benjumea, Francisco Gutierrez, Maria J Faus, Luis F Rodriguez

**Affiliations:** 1 Grupo Promoción y Prevención Farmacéutica, Facultad de Química Farmacéutica, Universidad de Antioquia UdeA, Calle 70 No 52-21, Medellín, Colombia; 2 Programa de Ofidismo y Escorpionismo, Facultad de Química Farmacéutica, Universidad de Antioquia UdeA, Calle 70 No 52-2, Medellín, Colombia; 3 Facultad de Farmacia, Universidad de Granada, Granada, España; 4 Orden Hospitalaria San Juan de Dios, Clínica San Juan de Dios, La Ceja, Antioquia, Colombia

**Keywords:** Bipolar disorders, Drug-related problems, Pharmaceutical care, Pharmacotherapy follow-up, Community pharmacy services, Pharmacists, Outpatients

## Abstract

**Background:**

Bipolar I disorder (BD-I) is a chronic mental illness characterized by the presence of one or more manic episodes, or both depressive and manic episodes, usually separated by asymptomatic intervals. Pharmacists can contribute to the management of BD-I, mainly with the use of effective and safe drugs, and improve the patient’s life quality through pharmaceutical care. Some studies have shown the effect of pharmaceutical care in the achievement of therapeutic goals in different illnesses; however, to our knowledge, there is a lack of randomized controlled trials designed to assess the effect of pharmacist intervention in patients with BD. The aim of this study is to assess the effectiveness of the Dader Method for pharmaceutical care in patients with BD-I.

**Methods/design:**

Randomized, controlled, prospective, single-center clinical trial with duration of 12 months will be performed to compare the effect of Dader Method of pharmaceutical care with the usual care process of patients in a psychiatric clinic. Patients diagnosed with BD-I aged between 18 and 65 years who have been discharged or referred from outpatients service of the San Juan de Dios Clinic (Antioquia, Colombia) will be included. Patients will be randomized into the intervention group who will receive pharmaceutical care provided by pharmacists working in collaboration with psychiatrists, or into the control group who will receive usual care and verbal-written counseling regarding BD. Study outcomes will be assessed at baseline and at 3, 6, 9, and 12 months after randomization. The primary outcome will be to measure the number of hospitalizations, emergency service consultations, and unscheduled outpatient visits. Effectiveness, safety, adherence, and quality of life will be assessed as secondary outcomes. Statistical analyses will be performed using two-tailed McNemar tests, Pearson chi-square tests, and Student’s t-tests; a *P* value <0.05 will be considered as statistically significant.

**Discussion:**

As far as we know, this is the first randomized controlled trial to assess the effect of the Dader Method for pharmaceutical care in patients with BD-I and it could generate valuable information and recommendations about the role of pharmacists in the improvement of therapeutic goals, solution of drug-related problems, and adherence.

**Trial registration:**

Registration number NCT01750255 on August 6, 2012. First patient randomized on 24 November 2011.

## Background

Mental illness is one of those medical conditions with difficult management and approaches due to characteristics related to the patient, such as insight of illness [1], therapeutic non-compliance, self-medication, inadequate family environment, poor social support, use and abuse of psychoactive drugs, and distrust of medical, pharmacological, and non-pharmacological therapy. Other circumstances that complicate the management of mental illnesses are the side effects and adverse reactions to drugs used in treatment. Hence, with the goal of contributing to controlling, reducing, and improving the current and future conditions of patients with any kind of mental disorder, there is a need for monitoring and following up on the outcomes obtained by psychiatrists using drug therapy [2]. In addition, these proposals should involve all phases of the health-disease process: education, mental health promotion, prevention, diagnosis, treatment, and rehabilitation of mental illness [2]. Mental disorders represent a high burden of disability, morbidity, and mortality worldwide [3].

Bipolar disorder (BD) is a known severe chronic mental illness. People with BD experience drastic alterations in mood and a decrease in labor and social performance [4]. It is manifested by the presence of both depressive and manic episodes, mixed or hypomanic episodes, usually separated by asymptomatic intervals. In BD-I there is at least one manic episode, in BD-II there are at least one hypomanic or mixed episode and a depressive episode [5]. According to the World Health Organization (WHO), BD ranks as the sixth leading cause of disability-adjusted life years (DALYs) worldwide among all diseases (for the population aged 15 to 44 years), and life expectancy is significantly reduced for those who suffer this condition. In addition, between 25% and 50% of patients attempt suicide and about 15% commit it [6]. The last article published based on a survey of 61,392 adults from 11 countries, found that the total lifetime prevalence of bipolar disorder spectrum was 2.4% worldwide, a combination of prevalence rates of 0.6% for BD-I, 0.4% for BD-II, and 1.4% for sub-threshold bipolar disorder. Twelve-month prevalences were 0.4% for BD-I, 0.3% for BD-II, 0.8% for subthreshold BD, and 1.5% for BD spectrum [7]. BD-I patients are more likely to have poor adherence included rapid cycling, suicide attempts, and current anxiety or alcohol use disorder. Additionally, a mental health study conducted in Colombia in 2003, found that the lifetime prevalence of BD-I is 1.8% (2.1 in men and 1.8 in women); and BD-II corresponds to 0.2% (0.1 in men and 0.2 in women). The bipolar spectrum (BD-I, BD-II, cyclothymia, and others not specified) varies from 3% to 6.5% [8].

The pharmacist can contribute to the management of this problem by working with the psychiatrist and the multidisciplinary team responsible for the patient’s care (psychologist, nurses, occupational therapist, social worker, and nutritionist, among others) [9,10]. The pharmacist should guide efforts to achieve and implement interventions to maximize drug therapy outcomes, such as informing the patient verbally and in writing about the proper use of medications, assessing and improving the therapeutic adherence, following the effectiveness and safety of pharmacotherapy, and giving feedback to the physicians responsible for the patient’s treatment. There are several studies that highlight the role of pharmacists in achieving therapeutic goals, and their inclusion on the health team improves the appropriate use of medication and treatment adherence. Likewise, there are some studies of patients with mental illness showing that pharmacists, through pharmaceutical care, may reduce suicide risk and minimize relapses [11-13].

Pharmaceutical care is a professional practice in which the pharmacist takes responsibility for a patient’s medication-related needs. This is achieved by detecting, preventing, and solving drug-related problems or negative clinical outcomes. Such processes should be carried out in collaboration with the patient and other healthcare professionals, aiming to achieve specific outcomes that improve the patient’s life quality [14]. The Dader Method is a tool to carry out pharmaceutical care, so pharmacists may achieve the greatest possible effectiveness and safety of pharmacotherapy, and, in collaboration with the healthcare team, the patient, and the patient’s family, improve the quality of life in outpatients diagnosed with BD-I [15-17]. The aim of the present study is to assess the effectiveness of the Dader Method for pharmaceutical care in reducing the use of healthcare services (number of hospitalizations, emergency consultations, and unscheduled outpatient consultations) and increasing the effectiveness and safety of treatment in patients with BD-I who are discharged from or referred by the outpatient service of the San Juan de Dios Clinic (SJDC).

## Methods/design

### Study design

A 12-month, randomized, controlled, prospective, single-center clinical trial will be performed to compare the effect of the Dader Method of pharmaceutical care with the usual care process of patients in a psychiatric clinic.

### Study setting

The study is conducted in outpatients with BD-I who attend psychiatric consultation in the Hospital Order San Juan de Dios, which belongs to the SJDC, an institution recognized for its dedication to mental illness care. The clinic has a psychiatric area with 115 beds and a weekly average of 90 consultations of outpatients with mental disorders, such as depression, anxiety disorders, bipolar disorders, panic disorders, hyperactivity, mental retardation, and schizophrenia. The clinic is located in La Ceja, a municipality in the east of Antioquia, an area historically linked to a high prevalence of mental disorders as a result of inbreeding, which determines the selection of individuals with predisposing genetic factors [18-20], and other situations like psychoactive substance use disorders, alcohol, violence, poverty, and stress.

### Study population

The researchers will recruit 200 volunteer patients diagnosed with BD-I, based on the Diagnostic and Statistical Manual of Mental Disorders, 4th edition (DSM-IV). BD-I patients are more likely to have poor adherence, rapid cycling, suicide attempts, and current anxiety or alcohol use disorder. Psychiatrists will refer patients who might meet the inclusion criteria to the pharmaceutical care program; subsequently, the pharmacist will evaluate whether each patient meets all the inclusion criteria and none of the exclusion criteria, and then invite the patient to participate in the study.

The inclusion criteria will be the following:

• Male or female, between 18 and 65 years of age, and diagnosed with BD-I based on the Diagnostic and Statistical Manual of Mental Disorders, 4th edition (DSM-IV), who have been discharged from or referred by the outpatient service of the SJDC.

• Living in Medellín, Sabaneta, Envigado, Itagüí, Bello, Copacabana, Girardota, Barbosa, Guarne, Rionegro, Marinilla, La Ceja, El Carmen de Viboral, El Retiro, San Vicente, La Unión, and El Santuario (Antioquia, Colombia).

The exclusion criteria will be the following:

• Patients with their first manic episode, schizoaffective disorder, BD-II, cyclothymia, and other bipolar spectrum disorders, personality disorders that seem like BD, and sociopathic disorder.

• Patients with epilepsy, infected with the HIV, chronic decompensated disease (blood pressure values above 180/110 mmHg, total cholesterol above 300 mg/dL, low density cholesterol greater than 160 mm/dL, hemoglobin A1c greater than 9%, oxygen saturation lower than 90%).

• Patients unable to comply with the protocol requirements, including severe alcohol and drug use.

• Pregnancy or breastfeeding.

• Patients with intellectual disability, presence of any cognitive impairment impeding the comprehension and signature of the informed consent form, or illiteracy.

• Patients who refuse to sign the informed consent form (consent must be obtained before any study-related procedures are conducted).

• Patients in electroconvulsive therapy.

### Patient recruitment and group assignment

The pharmacist will inform the potential candidates about the study, will ask for their authorization to participate, and their decision, whether they want to participate or not, will be registered. In the case of patients who agree to participate, the variables related to the objectives of the study will be assessed and when they meet the inclusion criteria and sign the informed consent form they will be randomized to one of the two groups (intervention or control group). Patients who do not meet any of the inclusion criteria will be informed about it, and the respective registration will be filed.

### Withdrawal or termination from study

According to the investigator’s judgment or when applicable, patients and their families should be informed of circumstances under which their participation may be terminated by the investigator without the subject’s consent. Patients and their families should also be informed of procedures for safe and orderly termination should they decide to withdraw from the study before it is completed. In addition, patients may leave the study at any time. Patient’s data concerning the reason for withdrawal before completing the treatment will be registered and archived for statistical evaluation. Specific reasons for withdrawal or termination from study are: (1) voluntary withdrawal from the patient; (2) intervention is harmful to the health of the patient; (3) patient’s death; (4) development of exclusion criteria during the study or other safety reasons; or (5) non-compliance of the protocol. The intention-to-treat principle will be kept at all times and all subjects will be analyzed according to their allocated treatment group.

### Study completion

One month before ending the program, patients and their families will be informed about the completion of the study when they have completed 12 months of treatment. For ethical considerations, all patients will be followed periodically by phone calls and further medical history review in the routine control appointment with the physician.

### Randomization

Patients will be assigned to the intervention or the control group through a computer-generated randomization - designed by a person external to the study and otherwise unrelated to it - using Microsoft ® Excel 2007. Participants will be assigned to the treatment groups in sequential order, and the randomization list will be strictly confidential (randomization list maintained off-site by the study coordinator, only one person outside the study knows it). The software generates a sequence of 500 random numbers without repetition (1 to 500), assigning a control group code to 250 numbers and an intervention group code to the other 250. Subsequently, the numbers generated are automatically sorted in ascending order to determine each patient’s allocation to one of the two study groups. Participants will be assigned to the intervention or the control group in sequential order once the study’s coordinator (the pharmacist) verifies fulfillment of inclusion criteria.

### Blinding

Blinding of participants and pharmacists is not possible because of the nature of the intervention.

#### *Control group: usual care (Routine dispensing), verbal and written education*

Because there is no blinding, there is no ‘placebo’ treatment. Patients who meet the inclusion criteria will be informed about the study and they will be registered after their signed authorization. The control group (patients and their families) will receive usual care as well as verbal and written information provided by the pharmacist (routine dispensing, including oral counseling regarding drugs). The written material is about mental health (MH) and BD, with information focusing on the importance of adhering to pharmacological and non-pharmacological interventions to achieve treatment goals. Randomization will take place during week zero (baseline) and patients will meet again with the pharmacist every three months (3, 6, 9, and 12 months). At each appointment, variables related to the primary (number of hospitalizations, emergency service consultations, unscheduled outpatient visits) and secondary outcomes (effectiveness, safety, adherence, and quality of life) will be assessed. In addition, patients will fill out a BD knowledge questionnaire every 3 months.

### Intervention design

#### *Intervention group: the Dader Method for pharmaceutical care*

**Figure 1:**
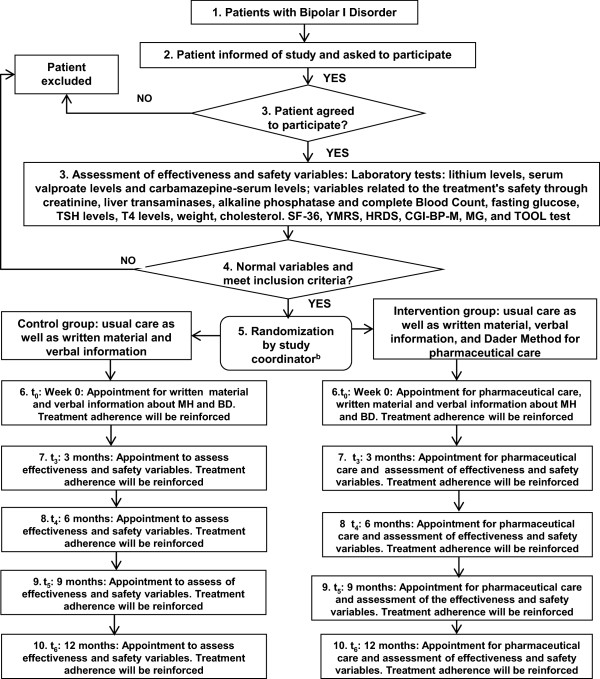
**General overview of EMDADER-TAB**^**a **^**study. **^a^EMDADER-TAB: Efecto del Método Dader de Seguimiento Farmacoterapéutico en pacientes con Trastorno Afectivo Bipolar I. (Effectiveness of Dader Method for pharmaceutical care in patients with bipolar I disorder]. ^b^The randomization takes place during week zero (baseline) and, during the first 20 days, the pharmacist will reinforce the information related to treatment adherence and ask the patient and his relatives for some criteria that will allow assessing treatment effectiveness and safety. In addition, the pharmacist will make two phone calls (week 1 and 3 from baseline) and a home visit (week 2 from baseline) to assess the variables related to the primary (number of hospitalizations, emergency service consultations, unscheduled outpatient visits) and secondary outcomes (effectiveness, safety, adherence, quality of life). Patients will meet again with the pharmacist every 3 months (3, 6, 9, and 12 months).

The Dader Method for pharmaceutical care is a systematic process developed by the Research Group of Pharmaceutical Care at the University of Granada, Spain [16]. The intervention is based on the use of pharmacotherapy records, evaluation of an assessment form that includes BD-I and the drugs used to treat this medical problem, and their assessment on a specific date. This assessment is used to identify: (1) any potential or actual patient health outcomes that are not consistent with the objectives of pharmacotherapy and are associated with the use of medicines (negative outcomes associated with medication (NOM)); and (2) situations in which the use of medicines caused or may cause the appearance of a NOM (drug-related problems (DRP)) [14]. Once the relevant problems are identified, the necessary interventions to patients or to physicians are carried out to solve the identified NOM and are followed by a subsequent assessment of the achieved outcomes. There is one pharmacist who is trained to perform the Dader Method of pharmacotherapy follow-up. Figure 1 shows the general study process. In the intervention group, the pharmacist is in charge of doing the following:

1. Developing the first assessment form: data related to mental medical problems are obtained by interviewing the patient, and using information from the drug and clinical records, prescriptions, other records (mainly drug history), and clinical laboratory tests that patients bring in the interview. Health problems (start date, whether it is controlled or not), drugs (start date, active principles, therapeutic strategy, dosage regimen and form of administration, previously used drugs, whether the patient uses the drugs or not, and knowledge of the drugs), patient expectations, lifestyle habits, and demographic data are registered in the assessment form, as well as other health problems with medical diagnosis. The data collected are used to complete the assessment form, which is interpreted and evaluated once all the necessary information is added. The key element to completing the assessment form is the pairing of health problems with the current drug therapy, which provides a global view of the patient’s health status and its relationship with the drugs used.

2. Evaluation and identification of suspected NOM: the collected information is used to study the health problems and drug treatment for subsequent evaluation, based on a literature review of the health problems and pharmacotherapy. The aim of this stage is to assess whether the desired treatment goals are achieved or not. The pharmacist must proceed with the overall patient’s assessment, in the identification and classification of suspected NOM or the possible risk of suffering from a DRP based on the conditions of need, effectiveness, and safety of drug therapy. For patients whose goals have not been achieved yet, the pharmacist will develop therapeutic plans that include interventions aiming to achieve the desired clinical outcome.

3. Intervention to prevent or resolve a NOM (intervention phase): this phase aims to resolve the NOM and DRP detected, and also to establish a monitoring plan to avoid the appearance of a new NOM. Once the pharmacist identifies concerns about the medical problems and current drug therapy, she/he interpreted and analyzed this information in the context of the clinical condition illustrated by the assessment form. If the aim of the intervention is to modify a problem with lifestyle or with the use of a medication, the recipient of the intervention is the patient or a relative. If the aim of the intervention is to modify drug therapy in a quantitative way (for example, modifying dosage or frequency) or in a qualitative way (for example, adding or changing any drug), the recipient of the intervention will be the physician. The intervention ends by verifying if the reason for the intervention has been solved or not and if enough time has been allowed to assess the intervention. The interdisciplinary teamwork should also be integrated in the intervention phase to combine efforts in treating the disease.

4. New assessment form of the patient: completion of an intervention should have generated a change in the patient’s assessment. The therapeutic plan is carried out depending on whether a NOM still exists or not, for example: informing the physician that mood stabilizers and antipsychotics medication are not effective and that the patient therefore may need a modification in drug therapy. Also, plan modifications are made when patients do not achieve positive results. In cases where the intervention needs to continue, an appropriate monitoring plan should be carried out according to the characteristics of the patient’s problems and idiosyncratic individuals’ situation.

Patients who meet the inclusion criteria will be informed of the study and they are registered after the authorization. In addition, the intervention group, patients and their families, will receive usual care as well as verbal and written information provided by the pharmacist (routine dispensing, including oral counseling regarding drugs). The written material is about MH and BD with information focusing on the importance of adhering to pharmacological and non-pharmacological interventions to achieve treatment goals. The randomization takes place during week zero (baseline), and, during the first 20 days, the pharmacist will reinforce the information related to treatment adherence and ask the patient and his relatives for some criteria that will allow assessing treatment effectiveness and safety. In addition, the pharmacist will make two phone calls (week 1 and 3 from baseline) and a home visit (week 2 from baseline) to assess the variables related to the primary (number of hospitalizations, emergency service consultations, unscheduled outpatient visits) and secondary outcomes (effectiveness, safety, adherence, quality of life). Patients will meet again with the pharmacist every three months (3, 6, 9, and 12 months). In addition, patients will fill a BD knowledge questionnaire every 3 months.

The intervention group patients will be monitored through the Dader Method for pharmaceutical care, over a 12-month follow-up period, to identify, prevent, and resolve drug-related problems with the process (causes) and drug-related problems with the outcome (effects). The strategy will consist of weekly phone calls and appointments with the patient, which, depending on the case, will take place at the patient’s home or at the pharmacist’s office. The pharmacist will inform the psychiatrist responsible for the patient in the case an effectiveness or safety problem related to the drugs is found, and the psychiatrist will determine the process to be followed.

### Study outcomes

#### *Evaluation/assessment variables/measuring instruments*

The primary outcomes to be measured are: (1) the number of hospitalizations; (2) the number of emergency service consultations; and (3) the number of unscheduled outpatient visits. In addition to the information provided by patients and family members about the primary objective, the pharmacist gets the information from the records of medical history of the patients, provided by SJDC.

The secondary outcomes to be measured are: (1) adherence to treatment through serum lithium levels, serum valproate levels, carbamazepine-serum levels, and by the questionnaire of Morinsky-Green (MG) [21]; (2) life quality through the Quality of Life Scale: The Short Form (36) Health Survey Questionnaire [22]; (3) Clinical Global Impression for Bipolar Modified scale, CGI-BP-M [23]; (4) Young Mania Rating Scale for the evaluation of mania [24]; (5) Hamilton Rating Scale for Depression [25]; (6) drug safety through creatinine, liver transaminases, alkaline phosphatase, complete blood count, fasting glucose, thyroid stimulating hormone levels (TSH), total T4 or total thyroxin levels (T4), weight and cholesterol; (7) problems related to necessity, effectiveness, and security of the pharmacotherapy (NOMs to be identified and measured); (8) drug-related problems in drug therapy effectiveness, and safety; (9) the impact of side effects of antipsychotic drugs on health-related quality of life with a specific self-rated instrument: the ‘Tolerability and quality of Life’ (TOOL) questionnaire [26]; and (10) the patient’s satisfaction with the pharmaceutical care service measured through the patient satisfaction questionnaire on pharmaceutical care [27]. Control and intervention groups will be compared in terms of the proportion of changes from baseline to follow-up at 12 months. The information collected at 3, 6, and 9 months is part of the follow-up period of patients, and these data help to complement the secondary outcomes of the trial. Secondary outcomes will handled in terms of the proportion of changes from baseline to 12 months following.

### Data collection

Data will be collected from November 2011 to June 2013. The study will have a total length of 30 months, but time monitoring of each patient will last 12 months. The recruitment period will be 18 months, and patients will be evaluated during 12 months, starting from the date of their recruitment. Once the enrolment period ends, the final 12 months will be only used to evaluate the latest patients. However, for ethical considerations, all patients will be followed periodically by phone calls and further medical history review in the routine control appointment with the physician. Monitoring and evaluation of the variables related to the study (including questionnaires) will take place as follows: at baseline, which corresponds to the day of study entry, and every 3 months at the patients’ check-ups to complete the year of follow-up. The data obtained in this study will be registered in an electronic database that will include the Morinsky-Green questionnaire to measure medication adherence, the scale SF-36 Health Status Questionnaire to estimate quality of life, the Clinical Global Impression for Bipolar Modified (CGI-BP-M) scale to measure the assessing global of illness, the Young Mania Rating Scale for evaluating mania, and the Hamilton Depression Rating Scale to measure depression. Data regarding medication history, interviews with the pharmacist, health current status, plans of action will be registered as well. Related to the primary outcome, in addition to the information provided by patients and family members, the pharmacist gets the information from the records of medical history of the patients, provided by SJDC.

### Data management

Data will be stored in a database Microsoft Access, 2007 with all patients’ information: details of medications and treatments, physical assessment records, laboratory tests, hospitalizations, and appointments. The database has an application that allows the use of psychiatric rating scales, and that information will also be incorporated in the database. The database shows reports of a patient’s assessment forms, monitor of the identified problems, and generates reports according to the user’s needs. The participant’s files and knowledge questionnaires are strictly confidential and will be archived in SJDC. Researchers involved in this study would be the only ones with access to the information. The pharmacist will perform a complete data backup every day, and the information will be stored in two different hard drives as a safety measure.

### Ethical issues

Institutional review boards that approved the study were: Faculty of Pharmaceutical Chemistry’s Research Technical Committee at Universidad de Antioquia, the SJDC’s Bioethics Committee (Reference Number: 06/10) and two healthcare providers’ Bioethics Committee: COOMEVA (Reference Number: 15/11) and SURA (Reference Number: 3/11). Each patient will receive verbal and written information about the study (purpose, procedures, potential risks, benefits, and information confidentiality), and also a copy of the informed consent form, which explains the study in detail. Once the patients resolve any doubts about the study, those who agree to participate must sign the informed consent form. The pharmacist must keep the original form and give a copy to the patient. The patient’s personal information (name, identification, address, age, gender, marital status, education, co-morbidities, prescription drugs) will never be disclosed at any phase of the clinical trial (final results, publications, or project presentations). The authors will guarantee publication of the outcomes of the clinical trial, and the results will be disseminated among the participants. A clinical monitor of the study will be reviewing the process constantly.

### Sample size calculation

The sample size and power were calculated using EpiInfo™ version 6.04 (Centers for Disease Control and Prevention, Atlanta, GA, USA). Assuming readmissions will be 50% in the control group and 30% in the intervention group, with a confidence of 95% and a power of 80%, it would be required to include at least 166 patients. The estimated sample size of 200 patients with a BD-I diagnosis is needed to cover losses during the follow-up and to increase sensitivity towards secondary objectives.

### Biostatistical considerations

Data will be analyzed according to the intention-to-treat principle. All patients will be analyzing according to their allocated treatment group. Statistical analysis will be blinded and performed using SPSS version 13.0 (IBM SPSS, Armonk, NY, USA). Control and intervention groups will be compared in terms of the proportion of changes from baseline to 12 months following. Repeated-measures analysis will carry out. Clinical assessments will be made at baseline and every 3 months during the study period. Follow-up will be continuing to complete the year, even if a relapse or psychiatric hospitalization occurred. The main end points of interest are the number and duration relapse events per person-year of follow-up and number of admissions to the hospital due to BD. Data will be reported as means and standard deviations (SD) or as percentages. The Pearson chi-square test (between study groups) and the McNemar test (within-group changes from baseline to follow-up at 12 months) will be used to compare proportions. Comparisons for categorical variables will be conducted by using Chi-square tests (the Fisher exact test when appropriate) and for continuous variables by using the independent-sample t test (Mann-Whitney U test when appropriate). The Mann-Whitney test will be used to compare outcomes between groups. For quality-of-life questionnaire the Student’s t test will be used if sample distribution proves to be normal. Otherwise, the Wilcoxon rank-sum test will be used. The paired Student’s t-test (within-group changes from baseline to follow-up at 12 months) or the independent sample Student’s t-test (between study groups) will be used to compare means; odds ratios (ORs) and 95% confidence intervals (CIs) will be estimated as well. Mean number and duration of relapse events normalized to person-time, will be compared between groups using the Student’s t test. Multivariable analyses will be performed to explain the association of multiple variables associated with the factors significantly related to primary outcome: hospitalizations, readmission, relapse and total number of episodes of illness, number and total days of hospitalizations, days of illness, and days of free intervals per year. The sociodemographic and clinical variables to assess will be: duration previous episodes, severity of depression or mania, polarity of the last episode (manic, hypomanic, depressive, or mixed), co-morbid psychiatric illness, social support, psychosocial factors, educational level, living in rural area, having a history of violence to others, having no insight, non-compliance to medication, lack of practical support from the family. Analysis of depression and mania episodes, adherence to pharmacotherapy and quality of life will be performed, using repeated measure analysis. Comparisons will be analyzed using two-tailed tests, *P* <0.05 will be considered as statistically significant.

### Missing data

According to Little and Rubin [28], the missing-data mechanism will be performed through the multiple imputation method, to replace missing values and to calculate accurate estimates of standard errors. The procedures all rest on the assumption that data are missing at random (MAR). We will include all non-missing values or outcomes at all time points and baseline demographics in the models that generated imputed estimates (that is, the likelihood of missing a follow-up visit can be predicted by available baseline or past observed data [29]). In addition, the multiple imputation method combines the likelihood-based analysis from each completed dataset is approximately equivalent to the analysis based on the observed-data likelihood, whereas the imputation uncertainty is reflected by the variation across the multiple completed datasets [30]. According to the definition of Fisher *et al.*[31], the intent-to-treat analysis will be include all randomized patients in the groups to which they were randomly assigned, regardless of their adherence with the entry criteria, regardless of the treatment they actually received, and regardless of subsequent withdrawal from treatment or deviation from the protocol [32]. The variable follow-up time for individual subjects, both the number of relapse events and duration of relapses during the follow-up will be calculated as the number (or duration (weeks)) of events per person per year of follow-up.

## Discussion

The final goal of the global mental health policy is to improve the quality of life of patients with mental illness. Pharmacists can contribute to this goal as members of healthcare teams performing pharmaceutical care, and therefore help optimize the mental health services and clinical management processes, through health promotion, and risk reduction of mental illness’ negative effects.

In Colombia, to our knowledge this is the first controlled trial designed to assess the effect of the Dader Method for pharmaceutical care on achievement of therapeutic goals in patients with BD-I (EMDADER-TAB). The primary goal of EMDADER-TAB is reducing the number of hospitalizations, number of emergency service consultations, and the number of unscheduled outpatient visits. The literature has established the impact of the pharmacist in the achievement of therapeutic goals in different diseases and conditions such as cardiovascular disease [33], asthma [34], depression [35], diabetes [36,37], and kidney transplants [38]; however, to our knowledge, there is a lack of randomized controlled trials designed to assess the effect of pharmacist intervention in patients with BD-I [12,39].

Through dispensing, pharmacotherapy follow-up, pharmacovigilance, and health education (pharmaceutical care components), the pharmacist contributes to achieve the best possible outcomes with drug therapy in a particular patient. Pharmacotherapy follow-up is done by identifying, preventing, and solving drug-related problems to improve the expected health outcomes [40]. This study will generate information about the effectiveness of the Dader Method for pharmaceutical care on the achievement of therapeutic goals in patients with BD-I, and will demonstrate the pharmacist’s role in the improvement of medication adherence, therapeutic outcomes, and the solution of drug-related problems in this group of patients. In addition, the study could generate some relevant contributions, both theoretical and practical, in the field of health-related services, as well as program formulations for pharmaceutical care in patients with BD-I. Thus, it will show that pharmacists, in association with the patients, their families, and the health teams, could contribute to achieve the therapeutic goals of BD-I.

The results could be subject to potential biases because: (1) the patients in this study will not be blinded to treatment assignment, and the same pharmacist, who is responsible for collecting trial data, will observe both the control and the intervention group; and (2) control group patients will receive phone calls from the pharmacist, who will encourage their attendance of appointments. These patients will also receive written material and verbal information about BD, MH, and counseling about the importance of adherence to pharmacological and non-pharmacological interventions to achieve therapeutic goals, as requested by the Human Research Ethical Committee in its approval of this research project. The effect of educational written material and the extra attention received by the patients in the usual care group might produce positive outcomes and reduce the magnitude of the differences with the intervention group.

## Trial status

This study is currently recruiting patients.

## Abbreviations

BD: Bipolar disorder; CGI-BP-M: The clinical global impression for bipolar modified; DSM-IV: Diagnostic and statistical manual of mental disorders, fourth edition; DRP: Drug-related problems; HIV: Human immunodeficiency virus; HRSD: Hamilton rating scale for depression; MH: Mental health; NOM: Negative outcomes associated with medication; QF: Quality of life; SJDC: San Juan de Dios Clinic - La Ceja, Antioquia, Colombia; T4: Total T4 or total thyroxine levels; TSH: Thyroid stimulating hormone; YMRS: Young mania rating scale.

## Competing interests

Authors declare they have do not have any potential conflicts of interests concerning the authorship or publication of this article. This research is been financed in part by Humax Pharmaceutical S.A., providing the PhD student with a salary and the written material used in this work. Data are being collected by ASO and will be interpreted by ASO, PA, and DMB. ASO, PA, and DMB will write the paper.

## Authors’ contributions

PA is the main investigator and he has developed the original idea for this research. The design of the study was done by PA, DMB, and ASO. FG has contributed to create the protocol and has contacted the healthcare providers. FG has also provided technical and medical assistance in the design of the study. MJF has also contributed to create the protocol and she is a member of the tutorial committee of PhD student. LFR contributed to the implementation of the project’s administrative activities performed at the clinic, such as: coordinating patient’s assignment to the pharmaceutical office, serving as a link with psychiatrists, and attaining physical space for the pharmaceutical office among others. Finally, all authors have corrected drafts and approved the final version of this study protocol.

## Authors’ information

ASO is PharmD and PhD student in Food and Pharmaceutical Sciences, University of Antioquia. PA is PhD, PharmD, and Professor at University of Antioquia, Medellin, Antioquia, Colombia. DMB is PhD, PharmD and Professor at University of Antioquia, Medellin, Antioquia, Colombia. FGˆ MD, SSc & MSc and Professor at University of Antioquia, Medellin, Antioquia, Colombia. MJF is PhD, PharmD, and Professor at University of Granada, Spain. LFR is MD and Medical Director of the SJDC.

## References

[B1] Ruiz MÁ Montes JM Correas Lauffer J Alvarez C Mauriño J de Dios Perrino C Opinions and beliefs of the Spanish population on serious mental illnesses (schizophrenia and bipolar disorder) Rev Psiquiatr Salud Ment 2012 5 98 106 Spanish 10.1016/j.rpsm.2012.01.002 22854580

[B2] Leclerc E Mansur RB Brietzke E Determinants of adherence to treatment in bipolar disorder: a comprehensive review J Affect Disord 2013 149 247 252 10.1016/j.jad.2013.01.036 23489403

[B3] Angst F Stassen HH Clayton PJ Angst J Mortality of patients with mood disorders: follow-up over 34-38 years J Affec Disord 2002 68 167 181 10.1016/S0165-0327(01)00377-9 12063145

[B4] Kessler RC Chiu WT Demler O Merikangas KR Walters EE Prevalence, severity, and comorbidity of 12-month DSM-IV disorders in the national comorbidity survey replication Arch Gen Psychiatry 2005 62 617 627 10.1001/archpsyc.62.6.617 15939839 PMC2847357

[B5] Fountoulakis KN Kasper S Andreassen O Blier P Okasha A Severus E Versiani M Tandon R Möller HJ Vieta E Efficacy of pharmacotherapy in bipolar disorder: a report by the WPA section on pharmacopsychiatry Eur Arch Psychiatry Clin Neurosci 2012 Suppl 1 1 48 22622948 10.1007/s00406-012-0323-x

[B6] Jamison KR Suicide and bipolar disorder J Clin Psychiatry 2000 61 47 10.4088/JCP.v61n0111 10826661

[B7] Merikangas KR Jin R He JP Kessler RC Lee S Sampson NA Viana MC Andrade LH Hu C Karam EG Ladea M Medina-Mora ME Ono Y Posada-Villa J Sagar R Wells JE Zarkov Z Prevalence and correlates of bipolar spectrum disorder in the world mental health survey initiative Arch Gen Psychiatry 2011 68 241 251 10.1001/archgenpsychiatry.2011.12 21383262 PMC3486639

[B8] Ministry of Social Protection National mental health study Colombia 2003 2005 Cali Colombia: Ministry of Social Protection and Social FES Foundation Spanish

[B9] Bell JS Rosen A Aslani P Whitehead P Chen TF Developing the role of pharmacists as members of community mental health teams: perspectives of pharmacists and mental health professionals Res Social Adm Pharm 2007 3 392 409 10.1016/j.sapharm.2006.10.005 18082875

[B10] Jones M Jones A Promotion of choice in the care of people with bipolar disorder: a mental health nursing perspective J Psychiatr Ment Health Nurs 2008 15 87 92 10.1111/j.1365-2850.2007.01208.x 18211555

[B11] Finley PR Crimson ML Rush AJ Evaluating the impact of pharmacists in mental health: a systematic review Pharmacotherapy 2003 23 1634 1644 10.1592/phco.23.15.1634.31952 14695043

[B12] Bell S McLachlan AJ Aslani P Whitehead P Chen TF Community pharmacy services to optimize the use of medications for mental illness: a systematic review Aust New Zealand Health Policy 2005 2 29 10.1186/1743-8462-2-29 16336646 PMC1345690

[B13] Gisev N Bell JS O'Reilly CL Rosen A Chen TF An expert panel assessment of comprehensive medication reviews for clients of community mental health teams Soc Psychiatry Psychiatr Epidemiol 2010 45 1071 1099 10.1007/s00127-009-0148-8 19826745

[B14] Committee of Consensus Third consensus of Granada on drug-related problems (DRP) and negative outcomes associated with medication (NOM) Ars Pharm 2007 48 5 17

[B15] Pharmaceutical Care Research Group University of Granada (Spain): dader method to provide pharmacotherapy follow up Ars Pharm 2005 46 309 337

[B16] Pharmaceutical Care Research Group, University of Granada (Spain) Pharmacotherapy follow-up: the dader method (3rd revision: 2005) Pharm Pract 2006 4 44 53 Spanish

[B17] Sabater Hernández D Silva Castro MM Faus MJ Dader method: guidelines for pharmacotherapy follow-up 2007 3 Granada: Pharmaceutical Care Research Group, University of Granada Spanish. [http://www.atencionfarmaceutica-ugr.es]

[B18] Ospina-Duque J Duque C Carvajal-Carmona L Ortiz-Barrientos D Soto I Pineda N Cuartas M Calle J Lopez C Ochoa L Garcia J Gomez J Agudelo A Lozano M Montoya G Ospina A Lopez M Gallo A Miranda A Serna L Montoya P Palacio C Bedoya G McCarthy M Reus V Freimer N Ruiz-Linares A An association study of bipolar mood disorder (type I) with the 5-HTTLPR serotonin transporter polymorphism in a human population isolate from Colombia Neurosci Lett 2000 292 199 202 10.1016/S0304-3940(00)01464-6 11018311

[B19] Ospina-Duque J Ochoa L García J López C Calle J Carvajal L Soto I Pineda N Duque C Miranda A Cuartas M Gómez J Agudelo A Montoya G Lopez M Gallo A Montoya P Palacio C Bedoya G Mccarthy M Reus V Freimer N Ruiz-Linares A Genetic loci associated to bipolar disorder: Studies in Columbian population: (Spanish) Rev Colomb Psiquiatr 2001 30 239 248

[B20] Bedoya G García J Montoya P Rojas W Amézquita ME Soto I Lopez MC Ospina-Duque J Ruiz-Linares A Isonymy analysis between 2 populations in northwestern Colombia: (Spanish) Biomedica 2006 26 538 545 17315480

[B21] Morisky DE Green LW Levine DM Concurrent and predictive validity of a self-reported measure of medication adherence Med Care 1986 24 67 74 10.1097/00005650-198601000-00007 3945130

[B22] Alonso J Prieto L Antó JM The Spanish version of the SF-36 health survey (the SF-36 health questionnaire): and instrument for measuring clinical results Med Clin (Barc) 1995 104 771 776 7783470

[B23] Spearing MK Pos RM Leverich GS Brandt D Nolen W Modification of the clinical global impressions (CGI) scale for use in bipolar illness (BP): the CGI-BP Psychiatry Res 1997 73 159 171 10.1016/S0165-1781(97)00123-6 9481807

[B24] Young RC Biggs JT Ziegler VE Meyer DA A rating scale for mania: reliability, validity and sensitivity Br J Psychiatry 1978 133 429 435 10.1192/bjp.133.5.429 728692

[B25] Hamilton M A rating scale for depression J Neurol Neurosurg Psychiat 1960 23 56 61 10.1136/jnnp.23.1.56 14399272 PMC495331

[B26] Montejo AL Lauffer JC Cuervo J Rebollo P Cordero L Diez T Maurino J Validation of a specific measure to assess health-related quality of life in patients with schizophrenia and bipolar disorder: the ‘tolerability and quality of life’ (TOOL) questionnaire Ann Gen Psychiatry 2011 10 6 10.1186/1744-859X-10-6 21396102 PMC3062605

[B27] Armando PD Vega EM Martínez-Pérez SR Martí-Pallarés M Solá NH Faus-Dáder MJ Validating a patient-satisfaction questionnaire about professional advice received for minor illness in community pharmacies Rev Salud Publica (Bogota) 2009 11 784 793 Spanish 20339604 10.1590/s0124-00642009000500011

[B28] Little RJA Rubin DB Statistical analysis with missing data 2002 2 New York City: John Wiley & Sons

[B29] Cheng AL Lin H Kasprow W Rosenheck RA Impact of supported housing on clinical outcomes: analysis of a randomized trial using multiple imputation technique J Nerv Ment Dis 2007 195 83 88 10.1097/01.nmd.0000252313.49043.f2 17220745 PMC3073142

[B30] He Y Missing data analysis using multiple imputation: getting to the heart of the matter Circ Cardiovasc Qual Outcomes 2010 3 98 105 10.1161/CIRCOUTCOMES.109.875658 20123676 PMC2818781

[B31] Fisher LD Dixon DO Herson J Frankowski RK Hearron MS Peace KE Peace KE Intention to treat in clinical trials Statistical issues in drug research and development 1990 New York: Marcel Dekker 331 350

[B32] Gupta SK Intention-to-treat concept: a review Perspect Clin Res 2011 2 109 112 10.4103/2229-3485.83221 21897887 PMC3159210

[B33] Amariles P Sabater-Hernández D García-Jiménez E Rodríguez-Chamorro MÁ Prats-Más R Marín-Magán F Galán-Ceballos JA Jiménez-Martín J Faus MJ Effectiveness of dader method for pharmaceutical care on control of blood pressure and total cholesterol in outpatients with cardiovascular disease or cardiovascular risk: EMDADER-CV randomized controlled trial J Manag Care Pharm 2012 18 311 323 22548691 10.18553/jmcp.2012.18.4.311 PMC10437626

[B34] Santos Dde O Martins MC Cipriano SL Pinto RM Cukier A Stelmach R Pharmaceutical care for patients with persistent asthma: assessment of treatment compliance and use of inhaled medications J Bras Pneumol 2010 36 14 22 20209303 10.1590/s1806-37132010000100005

[B35] Marques LA Galduróz JC Fernandes MR Oliveira CC Beijo LA Noto AR Assessment of the effectiveness of pharmacotherapy follow-up in patients treated for depression J Manag Care Pharm 2013 19 218 227 23537456 10.18553/jmcp.2013.19.3.218 PMC10438347

[B36] Fornos JA Andrés NF Andrés JC Guerra MM Egea B A pharmacotherapy follow-up program in patients with type-2 diabetes in community pharmacies in Spain Pharm World Sci 2006 28 65 72 10.1007/s11096-006-9003-0 16791717

[B37] Correr CJ Melchiors AC Fernandez-Llimos F Pontarolo R Effects of a pharmacotherapy follow-up in community pharmacies on type 2 diabetes patients in Brazil Int J ClinPharm 2011 33 273 280 10.1007/s11096-011-9493-2 21394570

[B38] Wang HY Chan AL Chen MT Liao CH Tian YF Effects of pharmaceutical care intervention by clinical pharmacists in renal transplant clinics Transplant Proc 2008 40 2319 2323 10.1016/j.transproceed.2008.06.050 18790223

[B39] Caley CF Bipolar disorder patient care opportunities: let’s answer the call Ann Pharmacother 2009 43 1890 1892 10.1345/aph.1M432 19809006

[B40] Faus MJ Amariles P Martínez F Pharmaceutical care: concepts, processes and studies 2007 Madrid, Spain: Ergon Spanish

